# Wheeled Mobility Use on Accessible Fixed-Route Transit: A Field Study in Environmental Docility

**DOI:** 10.3390/ijerph18062840

**Published:** 2021-03-10

**Authors:** Sol Lim, Clive D’Souza

**Affiliations:** 1 Department of Systems and Industrial Engineering, The University of Arizona, Tucson, AZ 85721-0020, USA; lims@arizona.edu; 2 Department of Industrial and Operations Engineering, University of Michigan, Ann Arbor, MI 48109-2117, USA

**Keywords:** environmental docility, wheeled mobility, accessibility, usability, public transit

## Abstract

Multiple field studies provide qualitative accounts of usability barriers experienced by users of wheeled mobility devices on public transit. This study aimed to examine these usability barriers from the theoretical perspective of Environmental Docility by quantifying the relationship between functional capabilities of wheeled mobility device users and ingress–egress performance on accessible fixed-route transit vehicles in an urban setting. Twenty-eight wheeled mobility users each completed three trips on a predetermined route through the local public transit system. Ingress and egress times, user-reported usability ratings and open-ended comments were analyzed. Regression analyses indicated significant interactions between age and minimum parallel-park length on ingress and egress times. Specifically, lower functional capability reflected in older age and less maneuvering ability predicted decreased performance (longer ingress–egress times), indicating less adaptability to environmental demands and agreement with the Environmental Docility Hypothesis. Usability ratings and comments revealed difficulty with negotiating access ramps and turning maneuvers in the vehicle interior and in proximity to other passengers. Despite compliance with accessibility standards, current design of transit vehicles present substantial usability barriers for wheeled mobility users. Environmental Docility provides a theoretical basis to identifying modifiable factors related to person and environment for improving usability of public transit for people aging and/or with mobility impairments.

## 1. Introduction

Accessible public transportation is vital to supporting community participation, positive health behaviors, and successful aging among people with disabilities [1,2,3]. In the US, the three decades since passage of the 1990 Americans with Disabilities Act (ADA) has witnessed an entire generation of people with disabilities participate in society raising the collective need and expectations for accessible public transit [4,5]. Despite progress, individuals with disabilities continue to experience barriers in public transportation impeding their access to community resources (e.g., healthcare, employment) and ability to socialize, risking social isolation particularly among adults aging with disabilities [2,6,7,8,9]. Demographic trends in disability and aging in the US and globally also contribute to the need for accessible public transportation [10,11]. Medical advances have increased longevity and survivorship of individuals with severe health conditions and traumatic injuries leading to more people aging with early-onset impairments and those aging into disability [2,12]. Aging increases the likelihood of acquiring a mobility impairment or other travel-limiting health condition [13]. For individuals unable to drive due to age or impairment, public transportation becomes an important mobility option for reducing dependence and social exclusion.

Users of wheeled mobility devices (e.g., manual wheelchairs, electric powered wheelchairs and scooters) in particular face unique design barriers when using fixed-route transit buses—the most prevalent mode of public transportation [14,15,16,17]. Urban transit buses in the US are predominantly large low-floor vehicles equipped with an electromechanical access ramp for a step-less ingress–egress and designated space for wheeled mobility devices [18,19]. These vehicles are considered accessible based on compliance with federal accessibility standards for transportation vehicles [20,21]. However, multiple studies describe issues with steep access ramps during ingress–egress and limited space for maneuvering a wheelchair on-board low-floor transit buses [14,16,17,19,22,23]. These barriers result in higher rates of anxiety and safety-critical incidents (e.g., accidental collisions, injuries) during ingress–egress on low-floor transit buses among wheeled mobility users compared to ambulatory passengers [24,25,26]. However, prior studies have lacked a theoretical foundation relating these user–environment interactions to intervention design and policy.

Problems related to accessibility for wheeled mobility users on fixed-route transit buses are not unique to the US [27,28,29]. Many transit providers are keen to increase fixed-route ridership by wheeled mobility users and other passengers with disabilities in order to address soaring costs of providing curb-to-curb special transportation services (e.g., complementary ADA paratransit in the US) [30,31]. Currently, the US has between 3.6 and 5.5 million community-dwelling individuals that use a wheeled mobility device, with numbers projected to increase by 5.2 to 8.8% annually [32,33,34]. Many countries including the UK, Canada and Australia are experiencing similar increases in the population of wheeled mobility device users [35,36,37,38].

### 1.1. Theoretical Context

This study was part of a research effort to operationalize relationships between functional capabilities of wheeled mobility device users and environmental barriers to community mobility including the use of public transit. The study was theoretically based in the ecological model of adaptation and aging [39,40]. Central to the ecological model is the notion that performance outcomes and adaptive behaviors result from a dynamic interaction between an individual’s impairment and the environmental context in which the individual is situated—a perspective that is shared by the International Classification of Functioning, Disability and Health definition of disability [41].

To characterize the interaction between person and environment, the ecological model introduces concepts of environmental demand or “press” exerted upon the person (e.g., from design features, resource constraints, social context), and functional capability or “competence” of the person encountering the environment. The model posits that by achieving a balance or fit between individual competence and environmental demand a user can be productively challenged by the environment and experience a sense of well-being as a consequence of behavior being adaptive [40]. Conversely, a mismatch or lack of fit in capability vs. demand manifests in reduced independence, increased physiological and psychological stress, and the disabling of users [42]. From this perspective, an accessible environment can be defined as an environment containing features or conditions—often codified in accessible design standards and guidelines—such that an individual with an impairment can ’function independently’ or with some minimally acceptable level of function to accomplish a goal-directed task [42,43]. Usability extends the definition of accessibility to also capture the quality of person–environment interaction in terms of efficiency (performance), effectiveness, and user satisfaction [44]. The Environmental Docility Hypothesis expands on the reciprocal capability-demand relationship by proposing that individuals with lower functional capacity are more vulnerable to environmental demands resulting in lower task performance compared to individuals with higher functional capability who are potentially less vulnerable to the effects of environmental demands [45].

### 1.2. Study Aim

The aim of this study was to quantify the relationship between functional capabilities of wheeled mobility device users (person component) and task performance (i.e., time duration) during ingress–egress on accessible low-floor public transit vehicles in a naturalistic fixed-route urban setting (environmental demand). Consistent with the Environmental Docility Hypothesis, we hypothesized that lower functional capabilities would be associated with lower performance (i.e., longer ingress and egress duration) under conditions of a moderately challenging environmental demand. Usability ratings of task difficulty and acceptability and open-ended comments were analyzed to understand and corroborate aggregate performance measures of ingress–egress times.

## 2. Materials and Methods

This study used a mixed-method approach consisting of demographic questionnaires and functional capacity assessments in an indoor laboratory, followed by a field-based ride-along technique that had participants independently complete 3 transit bus trips in succession through a predetermined route on the local public transit system. Two researchers shadowed the participant on the trips conducting a time-study and structured observations, and later administered post-trip ratings questionnaires. The study design was quasi-experimental implying that some study aspects were experimentally controlled (e.g., participant sampling within user group akin to a true experiment) or held constant (e.g., location and vehicle route, type of transit vehicle), while other aspects of the study were naturalistic and uncontrolled reflecting real-world conditions (e.g., in-service vehicles, bus schedules and timing, crowding levels, weather, bus driver).

### 2.1. Study Sample

Twenty-eight community-dwelling adults that regularly used wheeled mobility devices were recruited to participate in the study. They included 6 front-wheel drive powered wheelchair users (PWU), 11 mid-wheel drive PWU, 6 manual wheelchair users (MWU), and 5 scooter users (SU). Criteria for study inclusion required that participants rely on a wheeled mobility device as their primary means of mobility, be able to independently maneuver their mobility device, and use access ramps in buildings without assistance. Prior experience using public transit was not necessary, although participants were informed during recruitment that the study would entail riding public transit. In order to obtain a diverse sample, the research team recruited participants through multiple sources, including a local independent living center, the university community, advertisement postings in a local newsletter, and recruitment flyers posted at local senior centers, medical and rehabilitation centers, and public transit terminal. The university’s institutional review board approved the study procedures. All participants provided written informed consent prior to participation. The study was conducted with one participant at a time, and required between 2.5 and 3.0 h to complete with each participants receiving a US$75 honorarium.

### 2.2. Study Setting

**Figure 1:**
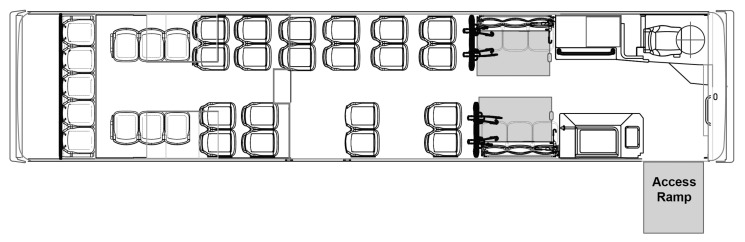
Plan view of interior configuration of the low-floor bus used in the study. The grey rectangles indicate the wheelchair access ramp at the front door for ingress–egress, and two forward-facing wheeled mobility securement areas in the bus interior.

The study was conducted in Ann Arbor, Michigan—a mid-western city in the US that is relatively flat in topography. The local transit service provider (i.e., the Ann Arbor Area Transportation Authority) provides fixed-route and complementary ADA paratransit services to the city and nearby townships. The transit provider has a fixed-route operational fleet consisting of 80 conventional 12 m (40-ft) length low-floor buses (Figure 1). Fares were paid on-board with prepaid fare-cards, cash, or coins. In an attempt to increase fixed-route ridership by people with disabilities, passengers eligible for the provider’s ADA paratransit service (e.g., those with qualifying medical conditions, or documented difficulty accessing/using the bus or bus stop) could travel on fixed-route buses for free.

The vehicles were compliant with prevailing federal accessibility standards [20,21]. Key features for wheeled mobility access included a kneeling feature to reduce the height difference from the curb, an electromechanical folding ramp at the front doorway that provides no-step access, an accessible pathway from the doorway to the vehicle interior. Each vehicle had two designated wheeled mobility securement areas (WMSAs; indicated using grey rectangles in Figure 1) located aft of the front wheel-well covers for situating passengers using wheeled mobility devices. Both areas had fold-up seats for other ambulatory passengers when the area was not occupied by a wheeled mobility user.

The WMSAs were each equipped with a four-point wheelchair tie-down system for securing the wheeled mobility devices in a forward-facing securement position. These are the most common type of wheeled mobility securement systems used on transit vehicles in the US. While federal standards mandate the provision of wheelchair tie-downs and occupant restraint systems (e.g., shoulder and lap belts), it permits individual transit service providers to establish their own policies regarding the use of these systems [20]. Hence, transit providers differ in their securement policies, some considering it mandatory while others give passengers in wheeled mobility devices the option for device securement [17]. The transit provider in our study required that passengers seated in wheeled mobility devices have their devices secured during travel, with the bus driver voluntarily assisting with the process of attaching and removing the four-point retractable tie-downs. Use of the safety restraint comprising a shoulder and lap belt system was optional at the discretion of the wheeled mobility occupant.

### 2.3. Study Procedure

#### 2.3.1. Pre-Trip Questionnaire Instruments and Functional Measurements

This first portion of the study was conducted at a private indoor facility close to the first bus stop. Participants were administered a questionnaire to obtain information about their age, gender, dominant hand, primary medical condition related to use of a wheeled mobility device, type of wheeled mobility device used, years of use, and the type and frequency of different travel modes used. Measurements of occupied mass (i.e., occupant and device combined) were obtained using a platform weighing scale. Occupied and unoccupied device length and width were measured using standard anthropometry calipers and tape measure.

Three additional measures of functional capacity were obtained. Power Grip Strength was recorded as the average of three measurements of 3 s maximal power grip exertions measured on the dominant hand using a Jamar grip dynamometer with the elbow flexed at $90^{\circ }$ with 2 min rest in between measurements [46,47]. Power grip strength provides an objective measure of upper extremity functional integrity relating to activities such as reaching, grasping, and wheelchair maneuvering, and is positively associated with improved quality of life [48,49,50].

Prior task analysis identified three types of wheelchair turns relevant to ingress–egress on transit vehicles, namely, a 90${}^{\circ }$ turn, 180${}^{\circ }$ turn, and a parallel park maneuver [51]. This study focused on the 180${}^{\circ }$ turn and parallel-park, which are more challenging to perform and require more space than the 90${}^{\circ }$ turn [52,53]. A 180${}^{\circ }$ turn width was measured as the minimum width required for completing a 180${}^{\circ }$ or U turn in a three-sided rectangular space constructed using movable lightweight cardboard walls. The measurement used a method of limits starting from a 1200 mm × 1200 mm space increased in 300 mm increments in width until successful [52,53]. Use of multiple short turns were allowed in contrast to a single continuous turn. Parallel park length was measured as the minimum space required for completing a left-hand side parallel park maneuver between two movable lightweight cardboard walls. The measurement used a method of limits. Starting from the participant’s occupied device length rounded up to the nearest 100 mm, the parallel park length between the walls was increased in 100 mm increments until the participant could successfully perform the parallel-park maneuver without contacting or displacing the walls [52].

#### 2.3.2. Transit Ride-Alongs and Trip Data

Next, each participant completed 3 bus trips in succession through a predefined route on the local transit system. Two researchers accompanied the participant. One researcher was responsible for providing the participant with instructions before the trip (e.g., the bus number to board and the stop to alight at), and administered a usability questionnaire after each bus trip (e.g., while at the bus stop awaiting the next bus). A second researcher performed direct observations and a time-study of the ingress–egress process using a handheld tablet PC. Data recorded for each trip also included: date, time of day, passenger load (i.e., the number of passengers on-board), number of passengers entering and/or exiting by their mobility status (i.e., ambulatory with no encumbrances; ambulatory with mobility aids such as cane or walker), and stop dwell-time. In order to minimize influence on ingress–egress times and potential observational bias, from the arrival of the bus until egress at the destination, both researchers assumed the role of independent passengers and avoided direct interaction or communication with the participant, i.e., fly-on-the-wall technique [54]. The researchers entered and/or exited after the participant and paid their fare using a swipe-card, thus had minimal effects on the participant’s ingress–egress time.

The general sequence of ingress tasks performed by the participant included: ramp ascent at the front doorway, moving to the WMSA, and entering and positioning in the WMSA. If both securement areas were vacant, the participant had the choice of using either one. Fare payment was not performed due to prior paratransit eligibility. All of the participants completed the bus trip while seated in their device and thus had their mobility device secured by the bus driver at the completion of ingress, and securement removed upon arriving at the destination. After securement removal, the sequence of egress tasks performed by the participant included: maneuvering out of the securement area, moving to the front doorway, and descending the access ramp. Participants were instructed to complete the ingress–egress tasks as they normally would, interacting with the bus driver when needed, but without acknowledging or communicating with the researchers.

#### 2.3.3. Route and Bus Stop Characteristics

The three bus trips undertaken were in a loop (i.e., start and end at the same stop adjacent to the indoor facility, Section 2.3.1), with average trip times (distances) of 8 min (3.2 km), 20 min (8 km), and 11 min (5.3 km), respectively. Participants alighted at the end of each trip, and waited for the bus taken on the subsequent trip to arrive. Depending on the arrival times of the preceding and subsequent bus and the time of day, the wait times at the intermediate bus stops were either 3 min or 18 min, and 17 min or 7 min, respectively. Thus, the total wait time was approximately either 20 min (3 + 17 min) or 25 min (18 + 7 min).

**Figure 2:**
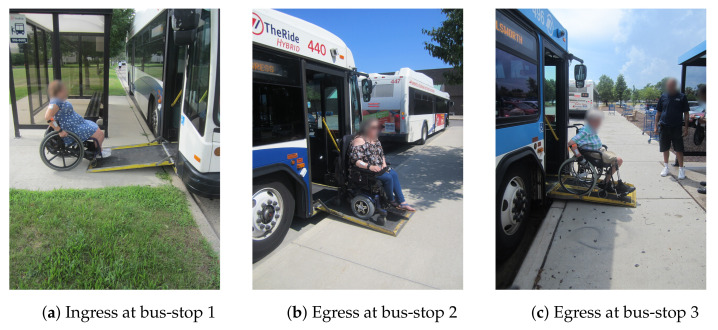
Images depicting a wheeled mobility device user at the three bus stops used in the study (**a**) Stop 1 at the start of Trip-1 and end of Trip-3, (**b**) Stop 2 used between Trips 1 and 2, and (**c**) Stop 3 used between Trips 2 and 3.

The three bus stops used in the study, depicted in Figure 2, complied with relevant federal accessibility standards [55]. Stop 1 was the least busy with the participant and both researchers being the only passengers entering or exiting at this stop on all study days. Stop 2 was located at a local shopping mall with a moderate turnover of between 3 and 10 passengers entering and/or exiting at the stop. Stop 3 was located in front of a large-chain grocery store and had busy pedestrian and vehicular traffic nearby compared to the other bus-stops. The data collection process concluded with the participant and both researchers returning to the indoor facility. Data collection only occurred on fair weather days, with an average ± SD temperature of $25.4\pm 4.4{\;}^{\circ }$ C across all study days.

### 2.4. Dependent Measures

#### 2.4.1. Task Duration

This study focused on time duration for 4 key tasks defined as follows:

- Ingress: starting from the instant the wheeled mobility device contacted the access ramp until the wheeled mobility user was positioned in the WMSA and either engaged the manual brakes or powered off the device.
- Securement attachment: starting from the end of the preceding task until the driver completed the device securement and started moving back to the driver workstation.
- Securement removal: starting from the instant the driver contacted the securement tie-downs for removal until the securement was removed, and the wheeled mobility user started operating or propelling the device towards the aisle.
- Egress: starting from the preceding task until the wheeled mobility user had descended the access ramp and the device was no longer in contact with the ramp.

Although device securement attachment and removal are performed by the bus driver, the corresponding times are reported since it contributes to dwell time associated with serving a wheeled mobility user [56,57].

#### 2.4.2. User-Reported Difficulty and Acceptability Ratings

After each bus trip (e.g., while awaiting arrival of the next bus) the participant was administered a questionnaire to rate the difficulty of seven tasks performed on the preceding bus trip using the Difficulty Rating Scale, namely, (1) moving to the vehicle access ramp at the bus stop, (2) ramp ascent, (3) moving to the WMSA, (4) entry and positioning in WMSA during ingress, (5) exiting the WMSA, (6) moving to the front door, and (7) ramp descent during egress. An overall rating of acceptability for the ingress–egress experience was obtained using the Acceptability Rating Scale.

The Difficulty and Acceptability Rating Scales are widely used measures of environmental usability and with established psychometric properties [25,58,59,60]. The Difficulty Rating Scale measures perceived ease or difficulty of task completion using a 7-point ordinal scale ranging from −3 (very difficult) to +3 (very easy). Respondents rate perceived task difficulty in two steps: (i) indicating if a completed task was “difficult”, “moderate”, or “easy”, and (ii) selecting a final rating from three possible options based on the general rating provide in the first step. Likewise, the Acceptability Rating Scale measures acceptability of a task in a two-step rating process using a 7-point ordinal scale ranging from −3 (very unacceptable) to +3 (very acceptable). For negative difficultly ratings, participants were asked to provide comments supporting their evaluations emphasizing barriers or constraints that may have impeded usability. The tasks of attachment and removal of wheeled mobility securement were not rated since these tasks were performed by the bus driver.

### 2.5. Statistical Data Analysis

Statistical analyses were performed using *R v.3.6.1* [61]. Summary statistics including means, standard deviations (SD), and ranges for continuous variables (i.e., age, structural anthropometry and functional capabilities), and frequency counts for categorical and ordinal variables (e.g., gender, years using mobility device, hand dominance, self-reported medical conditions and transportation modes) were computed for 4 user groups: front-wheel and mid-wheel drive PWU, MWU, and SU. Differences in age, anthropometry and functional capabilities between user groups were examined using one-way ANOVA at an a priori significance level of 0.10. Significant main effects of user group were examined using Bonferroni-adjusted pair-wise comparisons (p<0.10).

In order to minimize the effects of uncontrollable factors across bus trips (e.g., crowding levels, different bus drivers) that could influence participant behavior, the task times and user-reported ratings were averaged across the three bus trips for each participant for analysis purposes. One task time each from two participants were considered outliers and excluded from the analysis, namely, (i) Trip-2 ingress for one participant in a mid-wheel powered wheelchair was excessively delayed (267 s) due a different passenger in a wheelchair who was already on-board, and (ii) Trip-1 egress for one scooter user was delayed (263 s) due to the bus driver leaving the vehicle for a short while for reasons unknown to the researchers. Task times for these two participants were obtained by averaging the times from the remaining two trips. Average task times by user group were summarized graphically using box-plots.

Linear regression was used to test the study’s main objective, namely, examining the effects of 9 functional capability measures (i.e., standardized values for age, occupied mass, unoccupied and occupied length and width, maximum power grip strength, minimum parallel-park length, and minimum $180^{\circ }$ turn width) on ingress and egress times separately. Due to the small sample size relative to the number of potential predictor variables, a variable selection procedure that implements both forward and backward step-wise regression (in the R-package stats v3.6.1) was used to identify the reduced model with highest explained variance. Linear regression results representing separate reduced models for ingress and egress times with significant main and interaction effects were tabulated based on an a priori decision to infer statistical significance for values of p<0.10. Residual errors from both reduced models were examined separately using the Kolmogorov–Smirnov goodness of fit test with Lilliefors correction (p<0.05) as well as graphically using Q-Q plots, and confirmed that model assumptions of multivariate normality were satisfied. To help interpret the obtained reduced models, interaction plots were generated using input values at their mean ± 1SD (i.e., 0±1 for standardized variables) based on established practice [62,63].

To gain insight into differences in ingress–egress times, user-reported task difficulty and acceptability ratings were analyzed. Ratings for each participant were averaged over the 3 bus trips and summarized using medians and 90% confidence intervals (90% CI) stratified by user group [64]. The ratings were not normally distributed, hence separate nonparametric Kruskal–Wallis tests implemented with the R-package Rcompanion v2.3.7 [64] were used to examine differences in ingress–egress task ratings among user groups (front-wheel and mid-wheel PWU, MWU, SU). Effect size of the user group was calculated using the epsilon-squared statistic which assumes values between 0 (indicating no relationship) and 1 (perfect relationship) [65]. Effect sizes were interpreted as either small (between 0.01 and 0.08), medium (0.08 to 0.26), or large (≥0.26) based on criteria presented by Mangiafico [64]. Statistically significant effects of user group at *p*< 0.10 from the Kruskal–Wallis test were examined using pairwise comparisons [65] with the post hoc Dunn–Bonferroni test, and statistical significance was accepted at the Bonferroni-adjusted p<0.10 level. Lastly, responses from participants to prompts about barriers or constraints that may have impeded usability were aggregated by theme and summarized.

## 3. Results

### 3.1. Sample Demographics

Table 1 summarizes the demographic information of participants. The sample was 32% (n = 9) women, had a combined average ± SD age of 57.5 ± 15.6 years (including 10 participants aged 65+ years), and with 64% (n = 18) participants having used a wheeled mobility device for more than 10 years. Users of mid-wheel drive powered wheelchairs were slightly older than the other user groups, however age differences between user groups were not statistically significant (F(3,24)=1.25, p=0.315).

Participants reported a range of medical conditions, with the most frequent being the absence or loss of a lower extremity (17.9%), arthritis (14.3%), and orthopedic impairments (14.3%) (Table 2). Participants used different travel modes with varying frequency (Table 3). Over two-thirds of the sample used fixed-route buses (n = 20; 71%) and ADA paratransit (n = 18; 64%) at least once a month or more. Private or family-owned vehicles were used less frequently with 46% (n = 13 of 28) of participants using this transport mode once a month or more.

User groups differed significantly in occupied mass, power grip strength and minimum parallel park length (Table 4). On average, the occupied mass of front- and mid-wheel drive powered wheelchairs was significantly higher than manual wheelchairs and scooters. Power grip strength was significantly higher for MWU compared to front-wheel drive and mid-wheel drive PWU. Minimum parallel park length was significantly shorter for MWU compared to front-wheel and mid-wheel drive PWU and SU. Correlations between the different capability measures of age, anthropometry and maneuverability were generally low (|R|≤0.4). The few exceptions were minimum parallel park length being moderately correlated with occupied length (*R* = 0.66), occupied mass (*R* = 0.56), and grip strength (*R* = −0.56).

### 3.2. Ingress, Device Securement, and Egress Times

**Figure 3:**
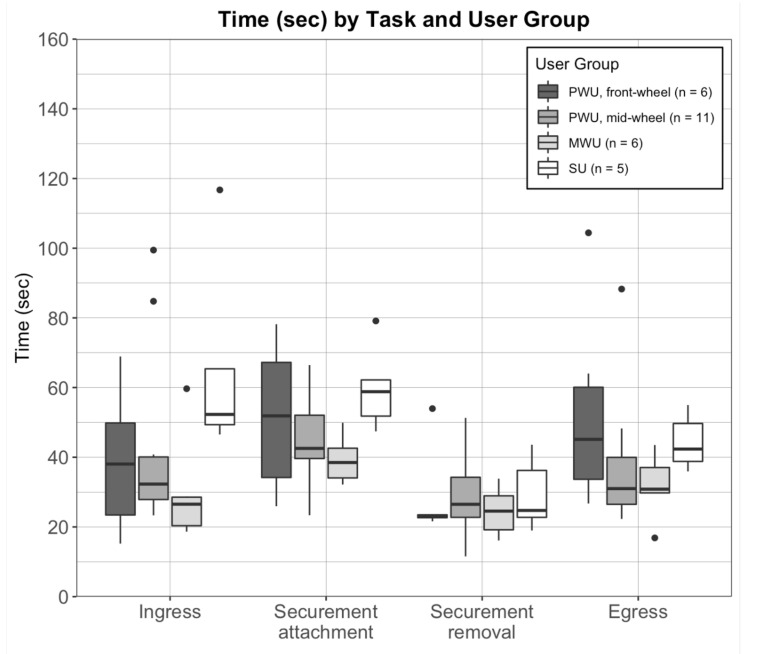
Box-plots of tasks times (seconds) for ingress, mobility device securement attachment and removal, and egress, averaged across 3 bus trips per participant and stratified by user group: mid-wheel (n = 6) and front-wheel (n = 11) drive powered wheelchair users (PWU), manual wheelchair users (MWU; n = 6), and scooter users (SU; n = 5). Dots indicate statistical outliers (i.e., values greater than 1.5 times the inter-quartile range).

Average ± SD times for ingress and egress were 43.1±25.0 s and 40.8±19.0 s, respectively. Average ± SD times for securement attachment and removal were 47.8±14.9 s and 27.7±10.1 s, respectively. Box-plots for ingress, securement attachment and removal by the bus driver, and egress times indicated high variability within user group (Figure 3).

**Figure 4:**
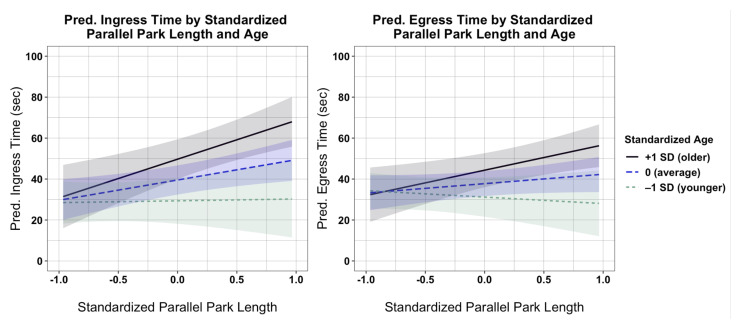
Interaction plots showing the effects of standardized parallel park length on predicted ingress (left panel) and egress (right panel) times at three values of standardized age: −1 SD (younger), 0 (average), and +1 SD (older). Error bands for each regression line represent 90% confidence intervals.

Regression analyses yielded reduced models with significant interaction effects of standardized age and parallel park length on ingress times $\left(F\left(3,24\right)=6.07,p=0.003;\mathrm{adj}.R^{2}\;=\;0.36\right)$, and on egress times $\left(F\left(3,24\right)=3.012,p=0.0498;\mathrm{adj}.R^{2}=0.18\right)$ (Table 5). Figure 4 depicts the simple slope of the regression of ingress time (left-panel) and egress time (right-panel) by standardized parallel park length for three age values: average (0), −1 SD younger, and +1 SD older. Ingress and egress times increased significantly with increases in age and parallel park length. Compared to an average ingress time of 29.6 s for users of average age and short parallel park length (−1 SD), a +1 SD increase in parallel park length was associated with an increase in ingress time by:

- +39.0 s (+132%) for older users (age: +1 SD);
- +19.8 s (+67%) for average-aged users (age: 0);
- Similar ingress times for younger users (age: −1 SD).

Likewise, compared to an average egress time of 33.2 s for users of average age and short parallel park length (−1 SD), a +1 SD long increase in parallel park length was associated with an increase in egress time by:

- +23.6 s (+71%) for older users (age: +1 SD);
- +9.2 s (+28%) for average-aged users (age: 0);
- A marginal −5.2 s (−16%) decrease in egress times for younger users (age: −1 SD).

### 3.3. Task Difficulty and Acceptability Ratings

**Figure 5:**
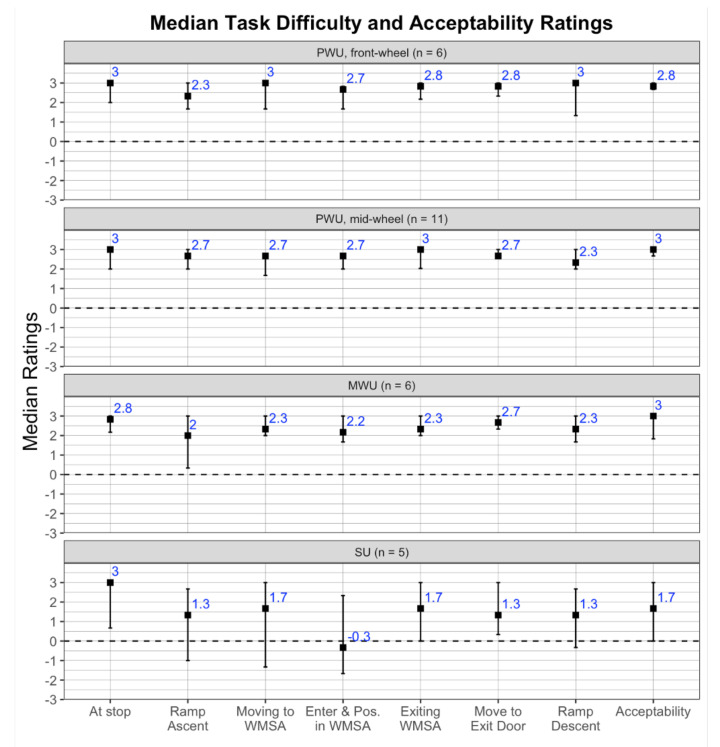
Medians (■) and 90% confidence intervals for task difficulty ratings (−3 = very difficult, 3 = very easy) and accessibility ratings (−3 = very unacceptable, 3 = very acceptable) averaged across 3 bus trips per participant and stratified by user group: mid-wheel (n = 6) and front-wheel (n = 11) drive powered wheelchair users (PWU), manual wheelchair users (MWU; n = 6), and scooter users (SU; n = 5).

Figure 5 depicts the median ± 90% CI for the task difficulty and overall acceptability ratings. Median task difficulty ratings for front- and mid-wheel drive PWU and MWU ranged between +2 (easy) and +3 (very easy), with the lowest ratings being for ramp ascent by MWU with a median of +2 (90% CI: +0.4, +3.0). With the exception of maneuvering at the bus-stop (median: +3), task difficulty ratings for SU were generally lower with medians ranging between +2 (easy) and −1 (moderately difficult). The task of entry and positioning in the WMSA had the lowest median rating (higher difficulty) of −0.3 (90% CI: −1.5, +2.4), with a significant difference among user group (p=0.063) (Table 6). Corresponding pair-wise post hoc tests indicated significantly lower difficulty ratings (greater difficulty) for SU compared to front-wheel drive PWU (p=0.099). None of the other comparisons were statistically significant. Overall acceptability ratings for the entire trip were slightly lower and more diverse for SU with a median of +1.7 (90% CI: 0.0, +3.0) compared to ratings for users of manual and powered wheelchairs (Figure 5), however, the differences were not statistically significant (Table 6).

### 3.4. Summary of Open-Ended Comments

Comments regarding ramp ascent concerned difficulty with turning at the top of the access ramp due to obstruction from the floor-mounted fare machine (one front-wheel, two mid-wheel drive PWU, one MWU, three SU), steep ramp slope (one mid-wheel PWU, three MWU), limited ramp width (three SU), and limited clearance space on the bus-stop when turning on to the access ramp (one mid-wheel PWU, one SU). Due to concerns about the ramp slope two participants (one mid-wheel PWU, one MWU) opted to ascend the ramp facing rear-wards as an adaptive strategy, while one MWU sought assistance from the bus driver with ramp ascent.

Remarks concerning moving to the WMSA were about the lack of choice in securement location either due to another wheelchair present on-board or the driver directing the participant to a specific location (two front-wheel drive PWU), limited space due to other passengers on-board with large items, e.g., a walker (one mid-wheel drive PWU), or general anxiety with the task anticipating difficulty with maneuvering in a constrained space (1 SU).

The related task of entry and positioning in the WMSA required performing a $180^{\circ }$ turn and parallel park maneuver into the securement area. Participants commented about the lack of turning space resulting in multiple turning maneuvers and stress due to perceived time pressure (one front-wheel drive PWU, three mid-wheel drive PWU, four SU). Two scooter users expressed preference for transferring out of their device to a seat after device securement but felt unsafe doing so due a lack of assistive handholds or stanchions for support. Two participants that ascended the ramp rearwards had to back into the WMSA without needing a $180^{\circ }$ turn, however the one mid-wheel PWU expressed safety concerns traversing in reverse while in close proximity to other seated passengers.

A few participants also expressed concerns about the device securement attachment and removal process. These comments referred to the inability of the bus driver to locate proper attachment points on the device and efficiently secure the device causing delays (one mid-wheel drive PWU, two MWU) and a perceived uncooperative attitude from the bus driver (one front-wheel drive PWU, one MWU, one SU).

Comments regarding exiting the WMSA concerned limited space for turning into the center aisle (one front-wheel and one mid-wheel drive PWU, one SU). Two participants also expressed difficulty reaching the stop cord (one front-wheel drive PWU, one SU) to indicate their destination stop. Moving to the exit door was challenged by limited aisle width near the front wheel-wells (one mid-wheel drive PWU) and safety concerns when other passengers are also exiting or entering the vehicle (one SU).

Remarks regarding ramp descent pertained to limited space for turning at the top of the ramp due to the obtrusive floor-mounted fare machine (one front-wheel and one mid-wheel drive PWU, four SU), limited ramp width (one front-wheel and two mid-wheel drive PWU, one MWU, one SU), steep ramp slope (one MWU), and limited runoff space on the bus-stop at the bottom of the ramp (one mid-wheel drive PWU, one SU). One participant requested verbal cues from the bus driver to help align with ramp edges prior to ramp descent.

## 4. Discussion

The Environmental Docility Hypothesis predicts that individuals with lower functional capability are more vulnerable to environmental demands resulting in lower performance compared to individuals with higher functional capability. Study findings support this hypothesis in the specific context of wheeled mobility device users during ingress–egress on low-floor public transit buses in a field setting. Importantly, task performance decreased (i.e., ingress and egress took significantly longer) for individuals with lower functional capability reflected in increased age and less maneuvering ability (i.e., longer parallel park length). User-reported task ratings and open-ended interview comments revealed difficulty with negotiating turning maneuvers and ramp gradients within the spatially constrained vehicle interior and when in close proximity to other passengers (Figure 5). These conditions led to experiences of heightened anxiety during ingress–egress, attitudinal barriers (e.g., uncooperative bus driver) and adopting potentially unsafe behaviors to overcome barriers (e.g., ascending the access ramp facing rear-wards, multiple three-point turns in close proximity to other passengers during entry-exit into the WMSA). Findings from this study corroborate prior studies that the current public transit environment, though compliant with accessibility regulations, presents substantial usability barriers for wheeled mobility users even in routine circumstances [25,51,66,67,68]. Prevailing conditions resulted in varied outcomes including potentially unsafe wheeled mobility maneuvers, reduced levels of independence, prolonged bus-stop dwell-times, and generally negative experiences for passengers using wheeled mobility devices that suggest a lack of person–environment fit and a potential disabling of this user group.

### 4.1. Theoretical Implications

Grounding this study in the Environmental Docility Hypothesis provides a theoretical perspective on the interactive relationship between person (operationalized using a set of functional capabilities) and environment (reflected in demands exerted by the environmental and social context) on behavior and/or performance outcomes. Environmental demand in this study was assumed to be of a moderate intensity—since the transit vehicles and bus stops were compliant with federal accessibility regulations, and a constant factor across participants—since relevant external factors were either similar (e.g., bus routes, vehicle and bus-stop design features, weather conditions) or averaged over multiple trips to minimize uncontrolled influences (e.g., number of passengers on-board and/or entering-exiting). Future studies could consider more detailed and objective measures to operationalize environmental demand across different types/designs of transit vehicles and bus-stops, transit service provider policies, and/or geographical location [69,70].

The person component was intentionally operationalized using functional capability measures rather than medical conditions or illnesses [71]. Compared to the overall population of wheeled mobility users, participants in this study could be considered relatively high functioning given that the inclusion criteria required ability to maneuver/propel and use access ramps independently in the community. Even in such a narrowly defined cohort of modest sample size, the nine functional capability measures related to age, anthropometry and space requirements for maneuvering revealed marked variability. Only three of these measures differed significantly by user group alone (Table 4). Findings reinforce the notion that wheeled mobility users are very heterogeneous with functional capabilities that lie on a continuous spectrum or gradient (vs. categorical). When challenged by moderate levels of environmental demands, this gradient in functional capabilities resulted in very different user experiences and performance outcomes.

Conceptualizing the disablement process as a dynamic interaction between an individual’s capabilities and the environmental context helps to identify modifiable factors for improving accessibility and usability of public transit and outcomes for individuals with disabilities. A consequence of the Environmental Docility Hypothesis is that behaviour and/or performance in activities relevant to daily living can be increased by enhancing functional capacity—the focus of rehabilitation practice, and/or by lowering environmental demand—often the emphasis of engineering design and policy. We discuss a few key implications of this study’s findings to both domains.

### 4.2. Implications for Rehabilitation

Knowledge of the relationships between functional capability and environmental demand are important when making decisions about a patients mobility needs. Example applications include when prescribing and customizing a wheeled mobility device to overcome specific functional deficits, when defining individual rehabilitation and functional training goals for wheeled mobility users, for evaluating an individual’s ability to use public transit, and/or documenting their eligibility for special transportation services such as ADA paratransit.

The significant interaction between age and maneuverability on task performance suggests a compounding of both these effects resulting in lowest performance for users that were older and had lower maneuvering capabilities. Younger and higher functioning individuals took significantly less time for ingress–egress suggesting greater adaptability to cope with the demands of transit vehicle ingress–egress, compared to their older and lower functioning counterparts. This finding corroborates qualitative studies that report poor mobility skills and lack of mobility confidence among older wheeled mobility users which severely limits their social participation [6]. The influence of age can potentially be explained by age-related functional declines in the physical, cognitive and perceptual domains [12,13]. For instance, sarcopenia or age-related declines in muscular strength and endurance is more prominent among people aging with disabilities, including users of wheeled mobility devices [72]. Older wheeled mobility users experiencing age-related changes also have a more negative evaluation of disability causing them to focus on disadvantages of their device, compared to their younger counterparts that view their device as empowering and enhancing social engagement [73].

Parallel park length as a measure of maneuverability is a combination of multiple modifiable factors related to the occupant and device jointly. Wheeled mobility devices support a broad range of health and functional needs stemming from diverse medical conditions [16,74]. Manual and powered wheelchairs are often medically prescribed and configured to the needs of individual consumers, including selecting appropriate device technology, weight-bearing capacity, device size (wheelbase, wheeltrack), body support/positioning (legrests and footrests, seat recline and/or tilt), and handling characteristics. Users of scooters tend to be older with some walking capability (e.g., at home) though need assistance with longer distances and/or outdoor mobility [16,75]. Collectively, these device options create a broad diversity in space requirements for accommodating occupied wheeled mobility devices and turning maneuvers [52,53,76,77]. Manual wheelchairs have a substantial maneuverability advantage during turning and terminal aiming (e.g., start-stop motions) tasks in constrained spaces compared to powered devices that can achieve faster speeds and better lateral control over long distances and/or larger width tolerances (e.g., wide open sidewalks) [78]. The drive wheel configuration for powered wheelchairs (e.g., front, mid, and rear) and scooters (3- vs. 4-wheeled) also influences its turn radius and related space requirement. Among the three drive configurations for powered wheelchairs, the mid-wheel drive is more maneuverable indoors due to a smaller $180^{\circ }$ turn and $360^{\circ }$ turn circumference while the rear-wheel drive is less maneuverable [52]. Overall, electric scooters have the largest turning radius and are least maneuverable of all wheeled mobility devices [53,79]. Study findings indicated similar trends with mid-wheel drive powered wheelchairs approaching manual wheelchair in average $180^{\circ }$ turn width but matching front-wheel drive powered wheelchairs and scooters in average parallel park length. Across all device types, users of scooters had the lowest median difficulty ratings for on-board circulation tasks and lowest overall acceptability ratings (Figure 5).

Clearly, the maneuverability of different mobility device types have implications for usability of the built environment (e.g., entering/exiting building, using public transit, using public restrooms) and overall community mobility. Multiple studies point to the trade-offs when using powered wheelchairs and scooters in terms of their ability to traverse longer distances versus challenges when maneuvering in confined spaces including compromised safety (e.g., higher rates of collisions causing personal injury, device damage, and injury to people nearby) [67,73,80,81]. Comorbidities such as vision and cognitive impairments could further impede safety and performance in device maneuvering tasks [82,83]. Wheelchair skills training for potential transit users would need to emphasize proficiency in tasks related to transit use, such as $180^{\circ }$ turns and parallel park maneuvers within constrained spaces and/or in close proximity to other people, and negotiating ascent-descent while turning on to narrow access ramps [84,85].

### 4.3. Implications for Accessible Public Transit

Article 9 of the UN Convention on the Rights of Persons with Disabilities requires that governments develop and implement standards to ensure accessibility of public transportation services [3]. Hence, our findings also have implications for countries looking to develop new standards and initiatives for accessible public transit. Regulations and standards for accessible design only prescribe minimum requirements, and thus cannot guarantee access for all wheeled mobility users. However, public transportation does carry the expectation that a substantial proportion of wheeled mobility users, for instance, higher functioning users represented in this study, can independently use public transit under routine conditions without compromising safety, dignity and/or satisfaction on par with their ambulatory counterparts. Findings from this study indicate recurring usability concerns with access ramps, limited on-board circulation space, and inadequate wheeled mobility securement area and equipment [19,27,76]. Steep gradients, inadequate edge barriers, and thresholds at the leading edge on access ramps also contribute to accidents and injuries among wheeled mobility users [22]. Current federal accessibility regulations for transit vehicles in the US permit a maximum slope of $9.5^{\circ }$ [20]. However, access ramp slopes vary considerably in field settings often exceeding the maximum permissible slope, with one study reporting ranges between $-4^{\circ }$ to $15.5^{\circ }$ [86]. Ramp ascent times, task difficulty, and perceived exertion are significantly higher for users of manual wheelchairs vs. powered devices [60]. Ramp descent on existing transit buses also poses usability issues, particularly for users of powered wheelchairs and scooters, due to the limited turn space at the front section of the bus (mostly from the floor-mounted fare payment device) leading to the ramp coupled with a narrow ramp width [25]. The interior seating configuration and location of the WMSA relative to the access ramp (e.g., front vs. middle of the bus) can greatly affect available floor space and ease of on-board circulation, more so in crowded conditions [25,51]. Current accessibility standards mandate that large transit vehicles have two designated spaces for wheeled mobility devices. One bus trip in this study had two wheeled mobility users on board, i.e., one user on-board while the study participant tried to enter the vehicle, which resulted in an ingress time approaching 4.5 min and was excluded from the analysis as an outlier. This particular scenario presents significant safety concerns in conventional low-floor bus designs, causes exceptionally long dwell-times and potential delays on routes that may be frequented by wheeled mobility users, e.g., routes serving hospitals, rehabilitation clinics [26].

The focus on ingress–egress times and its relationship to dwell-time also has operational and economic implications for public transit service providers. Delays in dwell-time and compromised passenger safety adversely impacts service quality for all riders [26,57]. Specific to wheeled mobility users, usability barriers and time delays discourage travel and/or increases reliance on more costly special transportation services [14,30]. Improving usability and accessibility in fixed-route transit potentially shifts riders to fixed-route, and reduces dependence on special transportation services with substantial financial savings for transit providers [31]. Universal design strategies to improving transit vehicle usability for wheeled mobility users (e.g., raised bus stop pads, increased interior circulation space) benefit many other riders groups including users of ambulation aids such as walkers, rollators, Segway’s, and users with luggage, push-strollers, or carrying small children, thereby increasing the overall population of potential transit riders [17,87,88,89].

### 4.4. Methodological Contributions

This study leveraged aspects of both field and laboratory settings. The study attained ecological validity by assessing task performance in participants’ natural transportation environment. Uncontrollable factors were either treated as a constant (e.g., vehicle design, route, bus-stops, narrow study window to minimize weather/seasonal effects) or averaged over multiple trials to minimize effects of factors such as crowding, bus driver, and bus stop conditions. Care was taken to only include task times that pertained to the wheeled mobility user using established procedures [56,89]. Task times obtained in this study were similar to corresponding times obtained in a previous naturalistic observational study of 15 wheeled mobility users, for instance, the average ± SD boarding (ingress + securement attachment) time obtained in this analysis was 90.9±33.1 s compared to a corresponding time of 84.3±24.2 s in the previous study [56]. Incorporating aspects of a controlled experiment in terms of participant selection and sampling, and detailed functional capability assessments in a lab-setting allowed for examining associations between specific person variables and task performance as opposed to relying on only observational data without direct participant interaction.

Findings from this study also emphasize the value of multi-method approaches to operationalize accessibility and usability in person–environment research. Generally, quantitative measures of task performance offer high validity, granularity (level of detail), and sensitivity to changes in capability and/or task demand. However, performance alone is an insufficient measure of usability [43,59]. The influence of psychosocial factors such as social interactions, time pressure, motivation, self-efficacy, and cultural norms on outcomes necessitates that usability evaluations incorporate perspectives of end-users [59]. Analyses of user-reported ratings and comments also supported this view. For example, study participants perceived themselves as delaying or inconveniencing others leading to heightened anxiety. The associated time pressure may cause them to rush and potentially cut corners on safety, i.e., a speed-accuracy trade-off. Maneuvering the wheelchair on-board the vehicle in close proximity to other passengers presents safety risks to the user and other passengers, particularly if the user lacks confidence in their maneuvering skills, or has a device that malfunctions and needs repair. At any given time, more than half of all wheeled mobility devices in the US are in need of repairs [90].

### 4.5. Study Limitations

The present study recruited a convenience sample of wheeled mobility users based on their self-reported ability to independently maneuver and use access ramps without assistance. Thus, the study sample is potentially biased toward more experienced and high functioning users, which may have led to underestimating ingress–egress times and user-reported difficulty relative to the general population of wheeled mobility users. Data on the proportion of all community-dwelling wheeled mobility users that meet these criteria and thus potentially capable of using fixed-route transit (e.g., candidates for fixed-route travel training) is not readily available.

This study’s sample size was relatively modest. Maneuvering capability (i.e., minimum parallel park length) and age were identified as significant predictors that explained the most variance in performance. This result should not be interpreted as other functional capability measures as being irrelevant, but only that they explained less model variance in a relative sense. In particular, parallel park length showed a moderate positive correlation with occupied mass and length, and a moderate negative correlation with power grip strength. While a larger sample would be beneficial in terms of statistical power and generalizability, the present sample was sufficient to yield initial evidence supporting the hypothesis and to identify key usability challenges associated with ingress–egress on fixed-route transit.

The study was limited to wheeled mobility ingress–egress on fixed-route transit vehicles. Hence, it presently did not include other aspects of the travel chain (e.g., route planning, going to/from the bus stop, navigating route transfers or large transit terminals) that present their own unique set of environmental barriers [5,28,29,91] or other users groups that are also vulnerable to transit barriers (e.g., individuals that use ambulation aids, or have vision, hearing and/or cognitive impairments) [88,92,93]. To maximize study participation, the study was conducted in the summer months under favorable weather conditions. Situations involving inclement weather such as snow and rain can render sidewalks inaccessible for ramp deployment or reduce traction on the surface of access ramps that further delays and/or impedes easy ingress–egress. In particular, snow introduces significant barriers to community mobility for wheeled mobility users, causing many to decrease or altogether avoid the use of public transit during winter [94].

## 5. Conclusions

This study uniquely operationalized the Environmental Docility Hypothesis in the specific context of wheeled mobility users’ ingress–egress performance on fixed-route transit vehicles. Device users of lower functional capability, reflected in older age and less maneuvering ability, took significantly longer time for ingress and egress, suggesting less adaptability to environmental demands compared to users with higher functional capabilities. User-reported task ratings and open-ended interview comments revealed difficulty with negotiating turning maneuvers and ramp gradients in the vehicle interior and in close proximity to other passengers.

Wheeled mobility devices are critical to supporting independence, autonomy, and social participation for people with severe mobility impairments. The ability to use public transit provides wheeled mobility users a cost-effective and reliable means of accessing community resources and successfully age in place. Despite decades of nationwide compliance with federal accessibility regulations in the US, the physical design of current transit vehicles still presents usability barriers for users of contemporary wheeled mobility devices. However, these usability barriers in public transit and the implications of this study are not limited to the US. Conceptualizing usability barriers from an ecological perspective, specifically the Environmental Docility Hypothesis demonstrated in this study, can help identify modifiable factors related to person (e.g., device type, maneuvering capability, grip strength) and environment (e.g., ramp design, circulation space) for improving accessibility and usability of public transit for people aging and/or with mobility impairments requiring wheeled mobility devices.

## Figures and Tables

**Table 1:** Demographic information of the study sample (n = 28) stratified by type of wheeled mobility device used. PWU = Powered Wheelchair Users, MWU = Manual Wheelchair Users, SU = Scooter Users.

Demographic Variable	Wheeled Mobility User Group				
	PWU, Front-Wheel (n = 6)	PWU, Mid-Wheel (n = 11)	MWU (n = 6)	SU (n = 5)	Total (n = 28)
Gender (Men, Women)	4, 2	9, 2	2, 4	4, 1	19, 9
Age, in years:
Mean ± SD	52.1 ± 19.6	64.5 ± 12.2	53.3 ± 17.0	53.8 ± 14.0	57.5 ± 15.6
Range (Min–Max)	29–75	38–83	31–71	37–69	29–75
Years Using Mobility Device:
1 months–5 years	1	4	1	-	6 (21%)
5–10 years	1	2	-	1	4 (14%)
>10 years	4	5	5	4	18 (64%)
Dominant Hand (Left, Right)	2, 4	9, 2	5, 1	4, 1	20, 8

**Table 2:** Primary medical condition reported by participants (n = 28) affecting physical mobility stratified by type of mobility device used.

Self-Reported Medical Condition	Wheeled Mobility User Group				
	PWU, Front-Wheel (n = 6)	PWU, Mid-Wheel (n = 11)	MWU (n = 6)	SU (n = 5)	Total (n = 28)
Absence or loss of lower extremity	-	3	2	-	5 (17.9%)
Arthritis	-	2	-	2	4 (14.3%)
Orthopedic impairment	3	1	-	-	4 (14.3%)
Cerebral palsy	-	2	-	1	3 (10.7%)
Neuromuscular degenerative disease	1	1	-	1	3 (10.7%)
Spinal cord injury	1	-	1	-	2 (7.1%)
Spina bifida	-	-	2	-	2 (7.1%)
Chronic Obstructive Pulmonary Disease	-	1	-	1	2 (7.1%)
Other ^1^	1	1	1	-	3 (10.7%)

^1^ One participant each with Multiple Sclerosis, Cardiovascular Disease, and Stroke, respectively.

**Table 3:** Self-reported frequency of using different transportation modes stratified by wheeled mobility user group (n = 28).

Transport Modes Used	Wheeled Mobility User Group				
	PWU, Front-Wheel (n = 6)	PWU, Mid-Wheel (n = 11)	MWU (n = 6)	SU (n = 5)	Total (n = 28)
*Fixed-route transit bus*	
≥once per week or more	4	6	2	3	15 (54%)
At least once per month	1	3	-	1	5 (18%)
≤once per month	-	1	2	1	4 (14%)
Never	1	1	2	-	4 (14%)
*ADA para-transit*	
≥once per week or more	4	6	1	2	13 (46%)
At least once per month	2	2	-	1	5 (18%)
≤once per month	-	1	3	2	6 (21%)
Never	-	2	2	-	4 (14%)
*Private or family-owned automobile or van*			
≥once per week or more	3	2	4	1	10 (36%)
At least once per month	-	1	-	2	3 (11%)
≤once per month	-	3	2	1	6 (21%)
Never	3	5	-	1	9 (32%)

**Table 4:** Mean ± SD values for occupied mass (kg), maximum power grip strength (N), unoccupied device length and width (mm), occupied length and width (mm), minimum parallel park length (mm), and minimum $180^{\circ }$ turn width (mm) stratified by wheeled mobility user group (n = 28).

Dimension	Wheeled Mobility User Group				
	PWU, Front-Wheel (n = 6)	PWU, Mid-Wheel (n = 11)	MWU (n = 6)	SU (n = 5)	Total (n = 28)
Occupied mass (kg) ***	277.0 ± 69.3 ^a,b^	234.9 ± 47.9 ^a,b^	96.6 ± 17.2 ^a^	142.1 ± 52.0 ^b^	197.6 ± 83.6
Max. power grip strength (N) **	91.0 ± 78.9 ^a^	93.8 ± 91.8 ^b^	253.0 ± 88.3 ^a,b^	118.7 ± 75.4	131.7 ± 104.0
Unoccupied device length (mm)	1127 ± 134	1020 ± 128	925 ± 55	1113 ± 150	1039 ± 138
Unoccupied device width (mm)	670 ± 73	72 ± 248	608 ± 33	621 ± 139	674 ± 171
Occupied length (mm)	1219 ± 113	1163 ± 154	1036 ± 112	1122 ± 149	1140 ± 144
Occupied width (mm)	758 ± 112	780 ± 229	608 ± 33	697 ± 134	724 ± 172
$180^{\circ }$ turn width (mm) ^1^	1417 ± 41	1245 ± 104	1200 ± 0	1360 ± 167	1293 ± 125
Parallel park length (mm) **	1400 ± 89 ^a^	1427 ± 156 ^b^	1117 ± 75 ^a,b,c^	1400 ± 187 ^c^	1350 ± 180

^1^ All manual wheelchair users completed the $180^{\circ }$ turn at 1200 mm. ^**^ p<0.05, ^***^ p<0.001. Letter superscripts indicate significant pairwise differences with Bonferroni adjustment for multiple comparisons at p<0.10.

**Table 5:** Summary results from the reduced linear regression models examining the main and interaction effects of standardized age and standardized parallel park length on ingress and egress times, respectively. Values in bold indicate significant effects at *p* < 0.10.

Parameter		Ingress Time (s)		Egress Time (s)	
	d.f.	Estimate (SE)	*F*, *p*-Value	Estimate (SE)	*F*, *p*-Value
Intercept	1, 24	39.5 (4.2)	9.51, **<0.001**	37.8 (3.6)	10.59, **<0.001**
Age	1, 24	10.2 (4.5)	2.26, **0.033**	6.6 (3.9)	1.70, 0.101
Parallel park length	1, 24	9.9 (4.2)	2.35, **0.028**	4.6 (3.6)	1.27, 0.216
Age × Parallel park length	1, 24	9.0 (4.4)	2.06, **0.050**	7.8 (3.8)	2.07, **0.049**

**Table 6:** Summary results from the non-parametric Kruskal–Wallis tests examining effects of user group (n = 28) on task difficulty ratings (−3 = very difficult; +3 = very easy) and overall acceptability rating (−3 = very unacceptable; +3 = very acceptable). Values in bold indicate significant effects at *p* < 0.10.

Dependent Variable	User Group	Effect Size (95% CI)
*Difficulty ratings by task*
1. At stop	$\chi^{2}$ = 1.21, *p* = 0.751	0.045 (0.014–0.395)
2. Ramp Ascent	$\chi^{2}$ = 4.31, *p* = 0.230	0.160 (0.044–0.520)
3. Moving to the WMSA	$\chi^{2}$ = 3.08, *p* = 0.379	0.114 (0.018–0.557)
4. Entry and position in the WMSA	$\chi^{2}$ = 7.30, *p* = **0.063**	0.270 (0.071–0.605)
	PWU, front-wheel > SU: Z = 2.40, adj. *p* = **0.099**	
5. Exiting the WMSA	$\chi^{2}$ = 2.24, *p* = 0.525	0.083 (0.018–0.552)
6. Moving to exit door	$\chi^{2}$ = 3.32, *p* = 0.345	0.123 (0.018 - 0.534)
7. Ramp descent	$\chi^{2}$ = 5.66, *p* = 0.130	0.210 (0.056–0.598)
*Overall acceptability rating*	$\chi^{2}$ = 1.75, *p* = 0.626	0.065 (0.013–0.474)

## Data Availability

The data presented in this study are available on request from the corresponding author. The data are not publicly shared for reasons of participant confidentiality.

## Abbreviations

The following abbreviations are used in this manuscript:

ADA	Americans with Disabilities Act
PWU	Powered Wheelchair Users
MWU	Manual Wheelchair Users
SU	Scooter Users
WMSA	Wheeled Mobility Securement Area

## References

[1] Bezyak J.L., Sabella S., Hammel J., McDonald K., Jones R.A., Barton D. (2020). Community participation and public transportation barriers experienced by people with disabilities. Disabil. Rehabil..

[2] LaPlante M.P. (2014). Key goals and indicators for successful aging of adults with early-onset disability. Disabil. Health J..

[3] United Nations Convention on the Rights of Persons with Disabilities: Article 9—Accessibility. https://www.un.org/development/desa/disabilities/convention-on-the-rights-of-persons-with-disabilities/article-9-accessibility.html.

[4] US Department of Justice The Americans with Disabilities Act of 1990 and Revised ADA Regulations (15 September 2010). https://www.ada.gov/2010_regs.htm.

[5] Steinfeld A., Maisel J.L., Steinfeld E. (2018). Accessible Public Transporation: Designing Service for Riders with Disabilities.

[6] Atoyebi O.A., Labbé D., Prescott M., Mahmood A., Routhier F., Miller W.C., Mortenson W.B. (2019). Mobility Challenges Among Older Adult Mobility Device Users. Curr. Geriatr. Rep..

[7] Syed S.T., Gerber B.S., Sharp L.K. (2013). Traveling towards disease: Transportation barriers to health care access. J. Community Health.

[8] Wolfe M.K., McDonald N.C., Holmes G.M. (2020). Transportation Barriers to Health Care in the United States: Findings from the National Health Interview Survey, 1997–2017. Am. J. Public Health.

[9] Henly M., Brucker D.L. (2019). Transportation patterns demonstrate inequalities in community participation for working-age Americans with disabilities. Transp. Res. Part A Policy Pract..

[10] Stevens A.C., Carroll D.D., Courtney-Long E.A., Zhang Q.C., Sloan M.L., Griffin-Blake S., Peacock G. (2016). Adults with One or More Functional Disabilities—United States, 2011–2014. Centers for Disease Control and Prevention, Washington, DC. Morb. Mortal. Wkly. Rep. MMWR.

[11] WHO (2011). World Report on Disability.

[12] Clarke P.J., Twardzik E., D’Souza C., Meade M., Lollar D.J., Horner-Johnson W., Froehlich-Grobe K. (2021). Chapter 11: Aging with a Disability. Public Health Perspectives on Disability—Science, Social Justice, Ethics, and Beyond.

[13] Okoro C.A., Hollis N.D., Cyrus A.C., Griffin-Blake S. (2016). Prevalence of Disabilities and Health Care Access by Disability Status and Type Among Adults—United States, 2016. Centers for Disease Control and Prevention, Washington, DC. Morb. Mortal. Wkly. Rep. MMWR.

[14] NCD (2005). The Current State of Transportation for People with Disabilities in the United States.

[15] NCD (2015). Transportation Update: Where We have Gone and What We have Learned.

[16] LaPlante M.P., Kaye H.S. (2010). Demographics and trends in wheeled mobility equipment use and accessibility in the community. Assist. Technol..

[17] Goldman J.M., Murray G. (2011). TCRP Synthesis 88: Strollers, Carts, and Other Large Items on Buses and Trains.

[18] King R.D. (1998). New Designs and Operating Experiences with Low-Floor Buses.

[19] Lenker J.A., D’Souza C., Paquet V., Steinfeld A., Maisel J.L., Steinfeld E. (2017). Vehicle Design (Chapter 6). Accessible Public Transportation: Designing Service for Riders with Disabilities.

[20] US Department of Transportation Federal Register 49 CFR Part 38—Americans with Disabilities Act (ADA) Accessibility Specifications for Transportation Vehicles. https://www.transit.dot.gov/regulations-and-guidance/civil-rights-ada/ada-regulations.

[21] US Access Board Updated ADA Accessibility Guidelines for Buses and Vans (14 December 2016). https://www.access-board.gov/ada/vehicles/update/buses-vans/.

[22] Frost K.L., Bertocci G., Smalley C. (2020). Ramps remain a barrier to safe wheelchair user transit bus ingress/egress. Disabil. Rehabil. Assist. Technol..

[23] D’Souza C., Zhu X., Steinfeld E., Lenker J., Paquet V., Mercario R.B. Human Factors Study of Accessible Buses Using Full Scale Simulations. Proceedings of the 14th International Conference on Mobility and Transport for Elderly and Disabled Persons (TRANSED), Part A.

[24] Frost K.L., Bertocci G., Smalley C. (2015). Ramp-related incidents involving wheeled mobility device users during transit bus boarding/alighting. Arch. Phys. Med. Rehabil..

[25] D’Souza C., Paquet V.L., Lenker J.A., Steinfeld E. (2019). Self-reported difficulty and preferences of wheeled mobility device users for simulated low-floor bus boarding, interior circulation and disembarking. Disabil. Rehabil. Assist. Technol..

[26] Kostyniuk L.P., D’Souza C. (2020). Effect of passenger encumbrance and mobility aid use on dwell time variability in low-floor transit vehicles. Transp. Res. Part A Policy Pract..

[27] Unsworth C., So M.H., Chua J., Gudimetla P., Naweed A. (2019). A systematic review of public transport accessibility for people using mobility devices. Disabil. Rehabil..

[28] Park J., Chowdhury S. (2018). Investigating the barriers in a typical journey by public transport users with disabilities. J. Transp. Health.

[29] Velho R. (2019). Transport accessibility for wheelchair users: A qualitative analysis of inclusion and health. Int. J. Transp. Sci. Technol..

[30] GAO (2012). ADA Paratransit Services: Demand Has Increased, but Little Is Known about Compliance.

[31] Thatcher R., Ferris C., Chia D., Purdy J., Ellis B., Hamby B., Quan J., Golden M. (2014). TCRP Report 163: Strategy Guide to Enable and Promote the Use of Fixed-Route Transit by People with Disabilities.

[32] Brault M. (2012). Americans with Disabilities: 2010.

[33] Taylor D.M. (2018). Americans with Disabilities: 2014.

[34] Flagg J., Bauer S., Buning M.E. (2009). Wheeled mobility demographics. Industry Profile on Wheeled Mobility.

[35] Smith E.M., Giesbrecht E.M., Mortenson W.B., Miller W.C. (2016). Prevalence of wheelchair and scooter use among community-dwelling Canadians. Phys. Ther..

[36] Barham P., Greenshields S., Mitchell J. (2020). Accessible Public Realm: Updating Guidance and Further Research, Technical Annex 2: A Review of the Dimensions of Wheeled Mobility Aids (RQ2), Project Report CPR2714. https://bit.ly/3mijBd4.

[37] Office of Fair Trading (2011). Mobility Aids: An OFT Market Study.

[38] Australian Bureau of Statistics, Government of Australia 2018 Disability, Ageing and Carers, Australia: Summary of Findings (Updated: 10 October 2019). https://www.abs.gov.au/statistics/health/disability/disability-ageing-and-carers-australia-summary-findings/2018.

[39] Lawton M.P., Nahemow L., Eisdorfer C., Lawton M.P. (1973). Ecology and the aging process. The Psychology of Adult Development and Aging.

[40] Nahemow L., Lawton M.P. (1973). Toward an ecological theory of adaptation and aging. Environmental Design Research.

[41] WHO (2001). International Classification of Functioning, Disability and Health (ICF).

[42] Steinfeld E., Danford G.S., Steinfeld E., Danford G.S. (1999). Theory as a basis for research on enabling environments. Enabling Environments.

[43] Iwarsson S., Ståhl A. (2003). Accessibility, usability and universal design—positioning and definition of concepts describing person–environment relationships. Disabil. Rehabil..

[44] ISO (2018). ISO 9241-11:2018: Ergonomics of Human-System Interaction—Part 11: Usability: Definitions and Concepts.

[45] Lawton M.P., Simon B. (1968). The ecology of social relationships in housing for the elderly. Gerontologist.

[46] Mathiowetz V., Weber K., Volland G., Kashman N. (1984). Reliability and validity of grip and pinch strength evaluations. J. Hand Surg..

[47] Fess E.E., Moran C.A. (1981). Grip and Pinch. Clinical Assessment Recommendations.

[48] Bohannon R.W. (2008). Hand-grip dynamometry predicts future outcomes in aging adults. J. Geriatr. Phys. Ther..

[49] Bohannon R.W. (1998). Hand-grip dynamometry provides a valid indication of upper extremity strength impairment in home care patients. J. Hand Ther..

[50] Sayer A.A., Syddall H.E., Martin H.J., Dennison E.M., Roberts H.C., Cooper C. (2006). Is grip strength associated with health-related quality of life? Findings from the Hertfordshire Cohort Study. Age Ageing.

[51] D’Souza C., Paquet V., Lenker J.A., Steinfeld E. (2017). Effects of transit bus interior configuration on performance of wheeled mobility users during simulated boarding and disembarking. Appl. Ergon..

[52] Koontz A.M., Brindle E.D., Kankipati P., Feathers D., Cooper R.A. (2010). Design features that affect the maneuverability of wheelchairs and scooters. Arch. Phys. Med. Rehabil..

[53] Steinfeld E., Maisel J., Feathers D., D’Souza C. (2010). Anthropometry and standards for wheeled mobility: An international comparison. Assist. Technol..

[54] Hanington B., Martin B. (2012). 42. Fly-on-the-Wall Observation. Universal Methods Of Design.

[55] US Access Board Americans with Disabilities Act Accessibility Standards, Section 810 Transportation Facilities (30 October 2006). https://www.access-board.gov/ada/#ada-810.

[56] Jayaprakash G., D’Souza C., Mercario R.B. Task Analysis Method to Modeling Wheeled Mobility User Ingress-Egress in Buses. Proceedings of the 14th International Conference on Mobility and Transport for Elderly and Disabled Persons (TRANSED), Part B.

[57] Kostyniuk L.P., D’Souza C. (2019). Dwell Time Models of Passenger Encumbrance and Mobility Aid Use on Low-floor Transit Vehicles. Proceedings of the 98th Annual Meeting of the Transportation Research Board.

[58] Danford G.S., Steinfeld E., Steinfeld E., Danford G.S. (1999). Measuring the influences of physical environments on the behaviors of people with impairments. Enabling Environments.

[59] Steinfeld E., Danford G.S. (2000). Measuring handicapping environments. J. Rehabil. Outcomes Meas..

[60] Lenker J.A., Damle U., D’Souza C., Paquet V., Mashtare T., Steinfeld E. (2016). Usability evaluation of access ramps in transit buses: Preliminary findings. J. Public Transp..

[61] R Core Team (2019). R: A Language and Environment for Statistical Computing.

[62] Aiken L.S., West S.G. (1991). Multiple Regression: Testing and Interpreting Interactions.

[63] Cohen J., Cohen P. (1983). Applied Multiple Regression: Correlation Analyses for The Behavioral Sciences.

[64] Mangiafico S.S. (2016). Summary and Analysis of Extension Program Evaluation in R, Version 1.18.1.

[65] Tomczak M., Tomczak E. (2014). The need to report effect size estimates revisited. An overview of some recommended measures of effect size. Trends Sport Sci..

[66] Unsworth C.A., Rawat V., Sullivan J., Tay R., Naweed A., Gudimetla P. (2019). “I’m very visible but seldom seen”: Consumer choice and use of mobility aids on public transport. Disabil. Rehabil. Assist. Technol..

[67] Mortenson W.B., Hammell K.W., Luts A., Soles C., Miller W.C. (2015). The power of power wheelchairs: Mobility choices of community-dwelling, older adults. Scand. J. Occup. Ther..

[68] Vredenburgh A.G., Zackowitz I.B. (2011). Research in Motion: A Case Study Evaluating the Accessibility of Public Transit in our Nation’s Capital. Proceedings of the Human Factors and Ergonomics Society Annual Meeting.

[69] Jensen G., Iwarsson S., Ståhl A. (2002). Theoretical understanding and methodological challenges in accessibility assessments, focusing the environmental component: An example from travel chains in urban public bus transport. Disabil. Rehabil..

[70] Iwarsson S., Jensen G., Ståhl A. (2000). Travel chain enabler: Development of a pilot instrument for assessment of urban public bus transport accessibility. Technol. Disabil..

[71] Carlsson G., Iwarsson S., Ståhl A. (2002). The personal component of accessibility at group level: Exploring the complexity of functional capacity. Scand. J. Occup. Ther..

[72] Maeda K., Shamoto H., Wakabayashi H., Akagi J. (2017). Sarcopenia is highly prevalent in older medical patients with mobility limitation: Comparisons according to ambulatory status. Nutr. Clin. Pract..

[73] Evans S., Frank A.O., Neophytou C., de Souza L. (2007). Older adults’ use of, and satisfaction with, electric powered indoor/outdoor wheelchairs. Age Ageing.

[74] Sprigle S., Chen J., Hughes D. (2020). Assessment of wheeled mobility devices provided to a commercially insured population in 2017. Assist. Technol..

[75] Isaacson M., Barkay D. (2020). Mobility scooters in urban environments: A research agenda. J. Transp. Health.

[76] Bharathy A., D’Souza C. (2018). Revisiting clear floor area requirements for wheeled mobility device users in public transportation. Transp. Res. Rec..

[77] D’Souza C., Steinfeld E., Paquet V., Feathers D. (2010). Space Requirements for Wheeled Mobility Devices in Public Transportation: An Analysis of Clear Floor Space Requirements. Transp. Res. Rec..

[78] Drury C., Koontz A., Feathers D., Kankipati P., Paquet V., Lin J.F. (2008). Controllability of Manual and Powered Wheelchairs for Spinal Cord Injury Users. Proceedings of the Human Factors and Ergonomics Society Annual Meeting.

[79] Dutta T., King E.C., Holliday P.J., Gorski S.M., Fernie G.R. (2011). Design of built environments to accommodate mobility scooter users: Part I. Disabil. Rehabil. Assist. Technol..

[80] Edwards K., McCluskey A. (2010). A survey of adult power wheelchair and scooter users. Disabil. Rehabil. Assist. Technol..

[81] Jang S., Mortenson B.W., Hurd L., Kirby R.L. (2020). Caught in-between: Tensions experienced by community mobility scooter users. Disabil. Soc..

[82] Massengale S., Folden D., McConnell P., Stratton L., Whitehead V. (2005). Effect of visual perception, visual function, cognition, and personality on power wheelchair use in adults. Assist. Technol..

[83] McMullan K.S., Butler M. (2019). Low vision and mobility scooters: The experiences of individuals with low vision who use mobility scooters. Disabil. Rehabil. Assist. Technol..

[84] Best K.L., Kirby R.L., Smith C., MacLeod D.A. (2005). Wheelchair skills training for community-based manual wheelchair users: A randomized controlled trial. Arch. Phys. Med. Rehabil..

[85] Mortenson W.B., Hurd Clarke L., Goldsmith C.H., Jang S., Kirby R.L. (2018). Measurement properties of the Wheelchair Skills Test for scooters among experienced users. Disabil. Rehabil. Assist. Technol..

[86] Bertocci G., Frost K., Smalley C. (2016). Public transit bus ramp slopes measured in situ. Disabil. Rehabil. Assist. Technol..

[87] D’Souza C., Paquet V., Lenker J., Steinfeld E., Bareria P. (2012). Low-floor bus design preferences of walking aid users during simulated boarding and alighting. Work.

[88] D’Souza C., Zhu X. (2014). Ambulation Aid Use and User Performance for Transit Vehicle Interior Design. Proceedings of the Human Factors and Ergonomics Society Annual Meeting.

[89] Kostyniuk L.P., D’Souza C. (2018). Encumbered passengers on fixed-route buses: An exploration. Proceedings of the 97th Annual Meeting of the Transportation Research Board.

[90] Toro M.L., Worobey L., Boninger M.L., Cooper R.A., Pearlman J. (2016). Type and frequency of reported wheelchair repairs and related adverse consequences among people with spinal cord injury. Arch. Phys. Med. Rehabil..

[91] Broome K., McKenna K., Fleming J., Worrall L. (2009). Bus use and older people: A literature review applying the Person–Environment–Occupation model in macro practice. Scand. J. Occup. Ther..

[92] Bareria P., D’Souza C., Lenker J., Paquet V., Steinfeld E. (2012). Performance of visually impaired users during simulated boarding and alighting on low-floor buses. Proceedings of the Human Factors and Ergonomics Society Annual Meeting.

[93] Crudden A., Cmar J.L., McDonnall M.C. (2017). Stress associated with transportation: A survey of persons with visual impairments. J. Vis. Impair. Blind..

[94] Ripat J., Sibley K.M., Giesbrecht E., Curtis B., Touchette A., Borisoff J., Ethans K., Li Y., Morales E. (2020). Winter mobility and community participation among people who use mobility devices: A scoping review. Arch. Rehabil. Res. Clin. Transl..

